# Opioid therapy vs. multimodal analgesia in head and neck Cancer (OPTIMAL-HN): study protocol for a randomized clinical trial

**DOI:** 10.1186/s12904-021-00735-0

**Published:** 2021-03-19

**Authors:** Sondos Zayed, Pencilla Lang, Lucas C. Mendez, Nancy Read, Jinka Sathya, Varagur Venkatesan, Dwight E. Moulin, Andrew Warner, David A. Palma

**Affiliations:** 1 Department of Radiation Oncology, London Health Sciences Centre, 800 Commissioners Road East, London, ON N6A 5W9 Canada; 2 Departments of Clinical Neurological Sciences and Oncology, London Health Sciences Centre, 800 Commissioners Road East, London, ON N6A 5W9 Canada

**Keywords:** Head and neck Cancer, Radiation Mucositis, Opioid analgesia, Multimodal analgesia, Radiotherapy, Randomized clinical trial, Non-inferiority

## Abstract

**Background:**

Radiation-induced mucositis (RIM) pain confers substantial morbidity for head and neck cancer (HNC) patients undergoing radiotherapy alone (RT) or chemoradiotherapy (CRT), often reducing treatment compliance. However, no standard currently exists for the treatment of RIM, and high dose opioid therapy, with its associated side effects and increased risk for chronic opioid use, remains the cornerstone of HNC pain management. The goal of this randomized clinical trial is to compare multimodal analgesia using analgesic medications with different mechanisms of action, to the institutional standard of opioid analgesia alone, in order to ascertain the optimal analgesic regimen for the management of RIM pain in HNC patients.

**Methods:**

In this open-label, single-institution, non-inferiority, randomized clinical trial, sixty-two patients with mucosal head and neck malignancies treated with curative-intent radiation will be randomized in a 1:1 ratio, stratified by RT or CRT, between Arm 1: opioid analgesia alone as per the institutional standard, or Arm 2: multimodal analgesia using Pregabalin, Acetaminophen, and Naproxen, in addition to opioids, if required. The primary endpoint is the average 11-Numeric Rating Scale (11-NRS) score for pain during the last week of radiation treatment. Secondary endpoints include: average weekly opioid use, duration of opioid requirement, average daily 11-NRS score for pain, average weekly opioids dispensed, quality of life, hospitalizations for analgesic medication-induced complications, time to feeding tube insertion, weight loss, toxicity, treatment interruptions, and death within 3 months of completing RT treatment. Patients are eligible once analgesia is required for moderate 4/10 pain.

**Discussion:**

This study will assess the efficacy and safety of multimodal analgesia and its impact on opioid requirements, clinical outcomes, and quality of life, as a potential new standard treatment for RIM pain in HNC patients undergoing definitive RT or CRT.

**Trial registration:**

ClinicalTrials.gov Identifier: NCT04221165. Date of registration: January 9, 2020. Appendix 2 reports the World Health Organization trial registration dataset.

**Supplementary Information:**

The online version contains supplementary material available at 10.1186/s12904-021-00735-0.

## Background

Patients diagnosed with head and neck malignancies experience substantial morbidity, largely due to pain. Head and neck cancer (HNC) pain is multifaceted. It may be attributed to the malignancy or to its treatment with chemotherapy, radiation, and/or surgery [1]. A significant proportion of patients (59–100%) undergoing radiotherapy alone (RT) or chemoradiotherapy (CRT) for their head and neck malignancy experience radiation-induced mucositis (RIM) or mucosal damage [2–4]. This often develops 2 to 3 weeks following the initiation of radiotherapy and worsens with dose accumulation throughout the course of their treatment. It is exquisitely painful and commonly persists for 2 to 3 weeks following treatment completion [2]. Although radiation to the head and neck is an effective oncologic treatment, patients often enter a vicious cycle of pain, dysphagia, aspiration, malnourishment, and reduced quality of life (QoL). This may translate into reduced treatment compliance, decreased oral intake requiring a feeding tube, hospitalizations and RT or chemotherapy treatment breaks, thereby reducing the chance of tumour control and cure [2, 5, 6]. Resource utilization, including admissions to hospital and other medical care costs have also been shown to increase in patients suffering from mucositis-related pain [7, 8]. Addressing head and neck RIM pain is therefore of critical importance to 1) maximize treatment compliance, 2) improve overall treatment outcomes, and 3) optimize healthcare resource utilization.

RIM pain comprises both nociceptive and neuropathic pain. Nociceptive pain encompasses both somatic pain – described as a well-localized sharp, throbbing pain – and visceral pain, characterized by its poorly-localized, dull nature. The nociceptive component of RIM pain is likely explained by radiation-induced direct mucosal injury, inflammation, and fibrosis over time. Nociceptive pain responds primarily to opioids, but also to Acetaminophen and nonsteroidal anti-inflammatory drugs (NSAIDs). Conversely, neuropathic pain is characterized by a burning, tingling sensation as a result of neural injury. The neuropathic pain component of head and neck mucositis may be attributed to tumour infiltration or radiation-induced polyneuropathy [5]. Gabapentinoids such as Gabapentin and Pregabalin are the mainstay of treatment for neuropathic pain [9]. RIM pain is therefore a multifactorial pain problem requiring a multimodal solution [1, 5].

Currently, opioid therapy remains the cornerstone of HNC pain management [3, 10–14]. In fact, patients with HNC have a higher prevalence of pain compared to other cancer types and this often translates into significantly higher rates of opioid prescription and substantially increases their risk for chronic opioid use [13, 15, 16]. Although effective for pain relief, opioids confer noteworthy morbidity in the form of nausea, vomiting, constipation, sedation, respiratory depression, hallucinations, tolerance, and dependence [10, 13]. An opioid crisis also looms over North America with thousands of lives being claimed to opioid misuse, addiction, and overdose [13]. Importantly, neuropathic pain does not respond effectively to opioid therapy and often requires escalating doses, thereby exacerbating opioid side effects [17–20].

We believe that adequate treatment of the two components of RIM pain – nociceptive and neuropathic – requires a multimodal analgesic approach [1]. Multimodal analgesia is defined as the treatment of pain using medications from different classes and different mechanisms of action. This may include regional anesthesia, opioid analgesia, systemic non-opioid analgesia, and adjuvants such as Gabapentinoids. Multimodal analgesia is now the foundation of the management of acute post-operative pain. Gabapentinoid, NSAIDs, and Acetaminophen use in the perioperative setting in multiple surgical specialties have been demonstrated to improve pain scores and decrease post-operative opioid use, without any significant increase in serious adverse events [21–23]. A similar multimodal analgesic approach has not been studied in HNC patients undergoing RT or CRT.

Gabapentinoids have been found to alleviate neuropathic pain in diabetic neuropathy, postherpetic neuralgia, trigeminal neuralgia, and post-operative pain [17, 18]. Few studies have explored the role Gabapentinoids play in treating radiation-induced neuropathic pain. A randomized double-blinded placebo-controlled clinical trial by Jiang et al. established that in HNC patients who suffered from chronic radiotherapy-related neuropathic pain after treatment, Pregabalin improved pain scores and quality of life compared with placebo [20]. Gabapentinoids have also been found to interact synergistically with opioids through the inhibition of nociception and simultaneous decrease in hyperexcitation [5]. Gabapentinoids such as Pregabalin therefore have the potential to alleviate the neuropathic component of RIM pain by potentiating the effect of opioids, thereby reducing the risk of escalating opioid requirements and opioid-related morbidity [5, 19].

NSAIDs may also play a role in decreasing the radiation-induced inflammatory response which contributes to mucositis pain [5, 10]. Synergy between NSAIDs and Acetaminophen as well as between NSAIDs and opioids would suggest that multimodal analgesia with these medications would likely reduce the total overall opioid consumption needed to achieve adequate pain relief while minimizing side effects [5, 19, 24, 25].

There is a paucity of evidence guiding the pharmacological management of RIM pain in HNC [2, 26, 27]. To our knowledge, this randomized trial will be the first study to assess multimodal analgesia as the optimal analgesic regimen for HNC patients undergoing radiation treatment.

## Methods

### Objectives

The objectives of the **Op**ioid **T**herapy vs. Mult**im**odal **A**na**l**gesia in **H**ead and **N**eck Cancer (OPTIMAL-HN) randomized clinical trial are to:

1. Determine whether multimodal analgesia using Pregabalin, Acetaminophen, and Naproxen, in addition to opioids, is non-inferior in terms of pain relief, to the institutional standard of opioid analgesia alone.
2. Compare the safety, efficacy, quality of life outcomes, toxicity, duration of and quantity of opioid use for both analgesic regimens.

Our hypothesis is that in HNC patients undergoing curative-intent RT or CRT, multimodal analgesia will be non-inferior to standard opioid analgesia alone for average pain scores during the last week of radiation treatment and will reduce the duration and quantity of opioid requirements.

### Study design

**Fig. 1:**
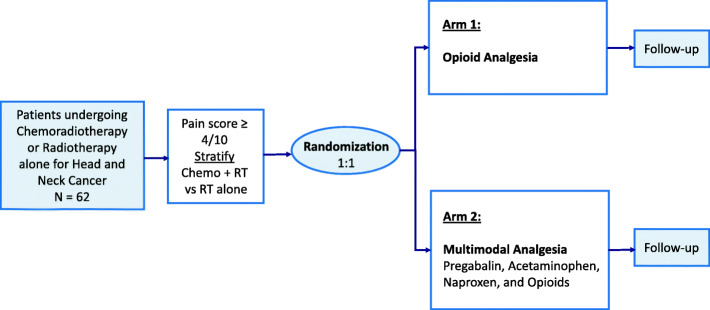
Study Schema. Abbreviations: RT – radiotherapy

This study is an open-label, single-institution, randomized clinical trial designed to assess the non-inferiority of two different analgesic regimens for RIM pain. The required sample size is 62 patients. Patients will be randomized to either opioid analgesia alone (Arm 1) or multimodal analgesia in the form of regular Pregabalin, Acetaminophen, and Naproxen in addition to opioids (Arm 2) in a 1:1 ratio, stratified by concurrent chemotherapy, once a threshold of moderate 4/10 pain is reached (Fig. 1).

#### Primary endpoint

- Average 11-Numeric Rating Scale (11-NRS) for Pain during last week of treatment
  - ◦ Defined as the average 11-NRS for pain documented daily during the last 7 days of the radiation treatment course

#### Secondary endpoints

- Average Weekly Opioid Use
  - ◦ Defined as the average weekly total opioid dose in oral morphine equivalent dosing (OMED) from date of randomization to 6 weeks after completion of radiation treatment
- Duration of Opioid Requirement
  - ◦ Defined as the time from the start of opioid treatment after date of randomization to the time of stopping opioid analgesia, in days
- Average Daily 11-NRS for Pain
  - ◦ Documented daily from date of randomization to 6 weeks after completion of radiation treatment
- Quality of Life (QoL)
  - ◦ Assessed with European Organisation for Research and Treatment of Cancer (EORTC) Quality of Life Questionnaire Core module (QLQ-C30) [28], and Head and Neck module (QLQ-HN43) [29], before radiation treatment, during the last week of radiation treatment, and 3 months after completion of radiation treatment
- Average Weekly Opioids Dispensed
  - ◦ Defined as the average weekly total opioid dose dispensed by the pharmacy in OMED from date of randomization to 6 weeks after completion of radiation treatment
- Admissions for any of the following, during radiation or within 3 months of radiation completion:
  - ◦ Febrile neutropenia
  - ◦ Other serious infection requiring treatment with intravenous antibiotics
  - ◦ Gastrointestinal bleeding attributed to NSAID use
  - ◦ Myocardial infarction
  - ◦ Stroke
  - ◦ Acute kidney injury defined by the KDIGO Guidelines [30] as:
    - ■ an increase in serum creatinine by ≥26.5 μmol/L within 48 h, OR
    - ■ an increase in serum creatinine to ≥1.5 times the baseline value which is known or presumed to have occurred within the prior 7 days
- Time to feeding tube (e.g. gastrostomy-tube or nasogastric-tube) insertion
  - ◦ Defined as time to feeding tube insertion after randomization, in days
- Weight loss from randomization to end of radiation treatment
- Rates of pre-specified Common Toxicity Criteria for Adverse Events (CTC-AE) toxicities
- Radiation or chemotherapy treatment interruptions
- Death during or within 3 months after completion of radiation treatment

#### Inclusion criteria

1. Age 18 years or older
2. Willing to provide consent
3. Histologically confirmed mucosal head and neck malignancy
4. Undergoing CRT or RT alone with a planned total radiation dose of 50 Gy or greater
5. Eastern Cooperative Oncology Group (ECOG) performance status 0–2
6. Life expectancy > 6 months
7. Onset of 4/10 pain on the 11-NRS that is localized to the mucosa of the mouth or throat, before or during radiation treatment, that is not caused by a current oral candidiasis infection.
8. Ability to take pills, either by mouth or crushed via nasogastric-tube or gastrostomy-tube
9. Ability to complete the study QoL questionnaires and pain diary
10. Ability to sign consent without requirement for a substitute decision maker

#### Exclusion criteria

1. Skin and salivary gland malignancies
2. High daily opioid use (defined as 30 mg oral morphine equivalent dose or higher) for more than 7 days at time of enrollment
3. Concurrent second active malignancy
4. Pregnant or lactating women
5. Psychological disorder requiring pharmacologic treatment
6. Regular systemic steroid use
7. Regular anticonvulsant, neuropathic or antidepressant use
8. Renal Impairment

- Defined as creatinine clearance < 60 mL/min

9. Liver Dysfunction

- Defined as total bilirubin > 34.2 μmol/L

10. Documented true allergy or contraindications to Acetaminophen, NSAIDs, Pregabalin, pantoprazole or opioids
11. History of upper gastrointestinal bleed
12. Known bleeding disorder
13. History of or current substance use disorder

#### Arm 1: opioid analgesia

The London Health Sciences Centre institutional standard for analgesic therapy in HNC patients undergoing RT or CRT is opioids, either with morphine or hydromorphone. Opioid analgesia for patients in Arm 1 will follow this institutional standard with initiation of opioids for moderate to severe pain, as per the World Health Organization (WHO) analgesic ladder. The use of Acetaminophen and NSAIDs in this arm will not be routinely recommended as that has not been the historic standard, due to concerns of masking a fever which could herald an infection.

Recommended opioid prescribing guidelines are as follows:

- Moderate pain (11-NRS Score 4–6; interferes significantly with activities of daily living (ADLs)):
- Low-dose morphine or hydromorphone may be prescribed at physician’s discretion.
- Severe pain (11-NRS Score 7–10; disabling, unable to perform ADLs):
- Regular strong opioids including morphine, oxycodone, hydromorphone, or fentanyl may be prescribed at physician’s discretion.
- Prescription of Methadone, Buprenorphine are discouraged since morphine dose equivalence is not well established for these opioids.
- Oxycodone/acetaminophen, codeine/acetaminophen or tramadol hydrochloride/ acetaminophen combination pills are also discouraged to limit the use of acetaminophen in this arm so as to prevent masking fevers.
- After initiation, the opioid dose may be titrated as necessary.
- An opioid rotation may be performed if either 1) opioid toxicity or 2) reduced analgesic efficacy despite dose escalation, are detected.
- Opioid toxicity may take the form of nausea, vomiting, constipation, sedation, respiratory depression, confusion, drowsiness, or hallucinations.
- A regular long-acting or regular short-acting opioid may be prescribed with breakthrough doses for transient pain exacerbations.
- The breakthrough dose should be calculated as 10% of the total 24-h opioid dose requirement.
- If more than 4–5 breakthrough doses are required daily for adequate pain relief, the baseline regular short-acting or long-acting opioid dose should be adjusted accordingly.
- The prescription of local analgesia in the form of creams or mouthwashes is left to the discretion of the treating physician since no clinically significant difference in pain relief has been observed between Doxepin Mouthwash or Diphenhydramine-Lidocaine-Antacid Mouthwash compared with placebo [31].
- Extended release opioid tablets may not be prescribed to feeding-tube dependent patients as crushing impedes their slow-release mechanism, thereby altering opioid absorption and efficacy.
- It is highly recommended to prescribe a concurrent laxative with opioids to reduce the morbidity of opioid-induced constipation. Patients will be provided with standard teaching materials on constipation management.

#### Arm 2: multimodal analgesia

Multimodal analgesia will be administered in Arm 2 with the **PAiN** Relief Regimen (**P**regabalin, **A**cetam**i**nophen, and **N**aproxen) in addition to opioids, if the latter become required.

- At the time of randomization, the following will be prescribed for analgesia using a standardized prescription template provided on the institutional electronic medical record:

1. Pregabalin:

- ▪ Step 1: 50 mg by mouth (PO) twice daily (BID) for 5 days
- ▪ Step 2: 100 mg PO BID for 5 days
- ▪ Step 3: 150 mg PO BID as a minimum maintenance dose if tolerated, until pain subsides
- ▪ If the patient has not attained adequate pain relief, the dose may be escalated to 200 mg PO BID for 5 days, 250 mg PO BID for 5 days, and 300 mg PO BID.
- ▪ Further dosage adjustments for tolerability and pain relief are to be titrated and optimized by the treating physician as needed. For example, the dose may be de-escalated to 25 mg or 50 mg PO BID if side effects occur.
- ▪ Taken regularly during radiation treatment until pain subsides
- Maximum dose: 600 mg/day

2) Acetaminophen

- ▪ 1000 mg PO thrice daily (TID) regularly (alternating with Naproxen) during radiation treatment and continued until pain subsides
- Patients will be advised to take their temperature before each dose, only if they feel unwell or if they experience any infectious symptoms
- Maximum dose: 3000 mg/day

3) Naproxen with concurrent Proton Pump Inhibitor (PPI)

- ▪ 250 or 500 mg PO BID regularly, titrated for pain relief (alternating with Acetaminophen, see Appendix 3) during radiation treatment and continued until pain subsides
- ▪ Concurrent Pantoprazole Magnesium 40 mg PO daily will be prescribed
- ▪ Patients will be counselled on signs and symptoms of gastrointestinal bleeding
- ▪ Patients will be advised to take their temperature before each dose, only if they feel unwell or if they experience any infectious symptoms
- ▪ Patients will be advised to take Naproxen with food
- ▪ Maximum dose: 1000 mg/day

For patients who become feeding-tube dependent, or are no longer able to swallow pills, all three medications can be taken as prescribed (crushed or capsule contents dissolved in water if necessary).

Opioids are to be initiated when pain control on this regimen is inadequate despite appropriate dose titration. The opioid prescribing guidelines outlined above will be adhered to in both arms.

Patients are encouraged to continue the PAiN relief regimen during opioid use to benefit from synergistic effects. However, if a patient is deriving no benefit from the PAiN relief regimen in the opinion of the treating physician, then they may continue on opioids alone.

### Data collection

REDCap will be used to complete the patient enrollment form and provide patient randomization information instantaneously in clinic. This will allow the patient to receive the appropriate prescriptions for analgesia at the same visit in order to alleviate their pain promptly. A hired research radiation therapist will enroll, randomize and collect the relevant data for study participants using the REDCap system.

Pain score data will be collected using the 11-NRS for pain. Patients will be asked to document average daily 11-NRS for pain in a pain diary which will be distributed at the time of enrollment and collected at the first follow-up visit, and 6 weeks after the completion of their RT treatment. Daily pain scores from the last 7 days of the radiation treatment course will be used to calculate the primary endpoint. This allows for capturing of pain scores when the patients have received most of the RT dose and when they are most symptomatic. Capturing data for several days takes pain variability into account and allows for a more accurate depiction of pain at the end of treatment. In order to ensure the preservation of pain score data, photocopies of the pain diary will be made for record-keeping purposes, every week, while the patient is receiving treatment.

Patients will also be asked to purchase study analgesic medications from the London Regional Cancer Program (LRCP) pharmacy only, in order to facilitate capture of total opioids dispensed as a secondary endpoint.

Patients will be instructed to bring in all study-related opioid medications on a weekly basis during their treatment, both empty and non-empty opioid bottles. The bottles indicate the name of the drug, the dose, as well as the total number of pills or volume dispensed for that specific bottle. This will all be noted for the empty opioid bottles and contents of empty opioid bottles will be considered consumed. Six weeks after the completion of RT, all remaining opioid bottles will be brought in by patients without exception, and the total opioid dose consumed based on empty bottles and remaining pills or opioid volume, will be recorded. The total opioid dose consumed by every patient from randomization until 6 weeks after the completion of radiotherapy will therefore be collected, tabulated, and converted into OMED, thereby allowing for the calculation of the secondary endpoint: average weekly opioid use. All excess unused opioids will immediately be returned to the patient once the data is collected. Patients will also be asked to indicate in the pain diary, the last day they required opioids for pain relief.

For missing data, the principle of the last observation carried forward will be applied whereby data from the previous week’s pain scores and analgesic drug use patterns will be substituted as an estimate.

Patient charts will be reviewed to document and capture hospitalizations, reasons for admission, and feeding tube insertions.

#### Consent process

Written informed consent will be obtained from all participants. A brief consent video will be shown to eligible patients, introducing them to the clinical trial and facilitating the informed consent process.

### Patient screening

Prominent signs will be placed in the LRCP patient review physician area indicating that if HNC patients have mucositis pain ≥4/10, to consider this trial, with contact information for study staff. Radiation therapists, nurses and nurse-practitioners caring for HNC patients are few with only a small rotation. They will be educated about the trial to facilitate candidate identification and accrual. During the consent process for radiation in the multi-disciplinary team clinic, the Clinical Specialist Radiation Therapist will mention the trial to patients to inform them of the option of the trial for pain management so they are aware they may be asked about it. If accrual stagnates despite this, the protocol will be amended to include signs in patients’ rooms in patient review stating: “Having pain because of head and neck radiation? Ask about the OPTIMAL trial.”

### Feasibility

To guard against accrual difficulties, the first six months of this protocol will be considered a feasibility phase. The study will proceed as designed during this phase, but monthly accrual totals will be emailed to the PIs. The accrual and randomization of the first 10 patients will be considered a feasibility phase. These first 10 patients must be enrolled within 6 months from the date of activation for the trial to be considered feasible to continue to full accrual. If this target is not met, the study investigators will meet with the Clinical Research Unit (CRU) leadership and make a joint decision as to whether the trial should be stopped, or if reasonable trial modifications could be instituted and another 6 months of accrual time be allowed to reach an accrual goal of 10 patients during that second period. Modifications which may be considered include: 1) a meeting before each patient review clinic with the nurses, nurse practitioners, and a clinical research coordinator to identify potentially eligible patients, and 2) a strategy meeting with head and neck radiation oncologists to further increase accrual. Patients participating in this clinical trial may also partake in other clinical trials if the trial protocol does not exclude patients enrolled in other trials.

### Adverse events [32]

#### Definitions of adverse events or reactions

***Adverse Event (AE)*** or reaction is defined as any unfavourable and unintended sign, symptom, abnormal laboratory finding, or disease temporally associated with the use of a medical treatment or procedure that may or may *not* be considered related to the treatment offered on trial.

***Serious Adverse Event (SAE)*** or reaction as includes any untoward medical occurrence that at any dose results in death, persistent or significant disability/incapacity, is life-threatening, or requires in-patient hospitalization or prolongation of existing hospitalization.

***Unexpected adverse reactions*** are of a nature and severity not consistent with the applicable prescribed medication in question, or occurs with more than expected frequency. Such reactions will be reported within 24 h.

All AEs, including the pre-specified AEs listed in Table 1, will be collected starting at randomization, captured during treatment and during the follow-up period, and 3 months after radiation treatment completion.

**Table 1:** Examples of treatment related adverse events

*Structure*	*Adverse Event*
Cardiac	Asystole
	Cardiac Arrest
	Myocardial Infarction
Gastrointestinal	Constipation
	Dry mouth
	Oral Mucositis
	Nausea
	Vomiting
	Lower gastrointestinal hemorrhage
	Upper gastrointestinal hemorrhage
	Duodenal hemorrhage
	Duodenal ulcer
	Duodenal perforation
	Dyspepsia
	Gastric hemorrhage
	Gastric ulcer
	Gastric perforation
	Colonic perforation
	Small bowel perforation
General disorders	Death NOS
	Localized edema e.g. peripheral or facial
	Fatigue
Investigations	Alanine aminotransferase increased
	Aspartate aminotransferase increased
	Alkaline phosphatase increased
	INR increased
	Prothrombin time prolonged
	Total, direct, or indirect bilirubin increased
	Hypoalbuminemia
	Creatinine increased
	Hyperkalemia
	Weight gain
Nervous system disorders	Cognitive disturbance
	Concentration impairment
	Depressed level of consciousness
	Lethargy
	Memory impairment
	Somnolence
	Stroke
	Transient ischemic attacks
	Dizziness
	Blurred vision
	Vision decreased
	Headache
	Ataxia
Psychiatric disorders	Confusion
	Delirium
	Hallucinations
	Insomnia
	Suicidal ideation
Renal and urinary disorders	Acute kidney injury
Skin disorder	Pruritis
	Dermatitis (either radiation-related or exfoliative)
	Stevens-Johnson Syndrome
	Toxic Epidermal Necrolysis
Immune system disorders	Anaphylaxis
Hematologic disorders	Anemia
	Agranulocytosis
	Thrombocytopenia
	Aplastic anemia
	Febrile neutropenia
Musculoskeletal and connective tissue disorders	Rhabdomyolysis

#### Definitions of causality

An adverse event or reaction is considered related to the research intervention (i.e. analgesic medications) if there is a reasonable possibility that the reaction or event may have been caused by the research intervention.

The relationship of an AE to the study treatment (causality) will be described as unrelated, unlikely, possible, probable, and definitely related. Definitions of each have been previously published [32].

#### Severity

The severity of AEs will be evaluated using the CTC-AE version 5.0 grading scale [33].

Grade 1: Mild.

Grade 2: Moderate.

Grade 3: Severe.

Grade 4: Life-threatening or disabling.

Grade 5: Death.

Note: The term “severe” is a measure of intensity of the symptoms, which may not necessarily be clinically concerning, as deemed by the treating physician.

#### Safety reporting

SAEs are to be reported using the SAE report form in REDCap. It is the responsibility of the Principal Investigator (PI) to report all SAEs to the REB as per local REB requirements. The PI should also comply with the applicable regulatory requirement(s) related to the reporting of **unexpected, related** serious adverse drug reactions to the Central Office within 24 h of discovery, and to regulatory authority (ies).

#### Data safety monitoring committee (DSMC)

Given that both arms of this trial represent treatment modalities that are in use as a standard at cancer centres worldwide, no DSMC will be used for this trial.

### Subject withdrawal

Subjects may voluntarily discontinue participation in the study at any time. Subjects removed from the study due to an adverse event should be observed by the treating physician at their discretion. All end-of-study investigations should also be obtained, whenever possible, provided the patient consents to do so.

### Follow-up evaluation

Patients will be seen in follow-up 6 ± 2 weeks and 3 months after radiation treatment completion. Requirements for weight measurement, 11-NRS pain score, analgesic use, and CTC-AE documentation, as well as QoL questionnaires, and laboratory investigations are outlined in Table 2 and Appendix 1. Patients will be asked to complete a pain diary (see Additional File 1) from the time of randomization until their 6-week follow-up appointment and to indicate in the pain diary the last day they consumed opioids. Opioid use documentation will also be performed weekly during radiation treatment, and at the 6-week follow-up appointment. At 3 months, patients will be asked whether or not they continue to require opioids for pain relief.

**Table 2:** Study Assessment Schedule

Assessments	At Randomization	During RT Treatment (weekly)	Follow-Up (6 weeks ± 2 weeks)	Follow-Up 3 months
**History and Physical**	X		X	X
**Weight Measurement**	X	X	X	X
**11-NRS Pain Score Documentation**	X	Daily Diary ^ǂ^	Daily Diary ^ǂ^	X
**Opioid Use Documentation**	X	X ^¥^	X ^¥^	X
**CTC-AE version 5.0**	X	X	X	X
**EORTC QLQ-C30 &** **QLQ-HN 43**	X	X Last week of RT treatment only		X
**Renal and Liver function assessment** (CBC, electrolytes, creatinine, AST, ALT, ALP, Bilirubin total & direct, INR, PTT, albumin)	X (≤ 7 days prior to randomization)	X Last week of RT treatment only (in addition to standard weekly bloodwork for patients receiving chemotherapy)	X	X
**Pregnancy Test for women of childbearing age**	X (≤ 7 days prior to randomization)			

RT: Radiation; 11-NRS: 11-Numeric Rating Scale

^ǂ^ From the time of randomization to the 6-week follow-up, patients will be asked to document daily average 11-NRS Pain scores

^¥^ On a weekly basis, pain diaries will be photocopied to ensure data are captured. Opioid use tabulation will take place weekly during radiation treatment by counting empty bottles (not counting pills at that time). The study staff may also meet with participants during a different daily visit for radiation if scheduling does not permit during the weekly Patient Review (PR) clinic visit. At the first follow-up appointment at 6 weeks, the pain diary will be collected, empty bottles will be tabulated, and if there are non-empty bottles, the pills used from those bottles will be counted. At the 3-month follow-up appointment, participants will be asked whether they continue to consume opioids for head and neck mucosal pain

Laboratory investigations will be performed at the time of randomization, once during the last week of radiation treatment, once at the 6 to 8-week follow-up and once at the 3-month follow-up appointment. The requirement for further laboratory investigations will be left at the discretion of the treating physician and will be dictated by the patient’s clinical state. For baseline laboratory investigations, values available within 7 days of the day of randomization may be used if available.

### Statistical considerations

#### Randomization

The study will employ a 1:1 randomization between Arm 1: Arm 2, stratified by use of concurrent chemotherapy (chemoradiotherapy vs. radiotherapy alone). A permuted block design with one stratification factor for concurrent chemotherapy will be used with the size of the blocks known only to the statistician. Randomization will be performed on the day of enrollment using REDCap.

#### Sample size calculation

The primary endpoint is defined as the average 11-NRS daily pain score during the last 7 days of the radiation treatment course. We assume a non-inferiority margin of 1 point on the 11-NRS for pain and a standard deviation of 1.5 in both arms. Using a two-sample T-test for non-inferiority, a one-sided alpha of 0.05, 80% power, and an estimated dropout rate of 6%, a total of 62 patients will be required to power this trial (31 patients in each arm).

#### Accrual target

The study projects accrual over 24 months, with 3 months of additional follow-up. Accrual targets are as follows: 31 patients per year.

#### Statistical analysis plan

In order to assess the sample size calculation assumptions, one interim analysis will take place after half of the patients have been accrued and have completed their 6-week follow-up visit (*n* = 31). At this analysis, the PIs will be blinded to the identity of each treatment arm and provided with the average daily 11-NRS for pain during the last 7 days of treatment and the standard deviation, for each arm. If the standard deviation differs substantially from the assumed standard deviation in the sample size calculation, the sample size may be increased or decreased at the discretion of the PIs, in order to maintain statistical power for non-inferiority between arms. No statistical comparisons will take place at the interim analysis.

Since this is a non-inferiority trial, the primary endpoint will be a per-protocol analysis. An intention-to-treat analysis will also be provided as a sensitivity analysis.

##### Primary endpoint

The average 11-NRS for pain during last week of treatment will be defined as the average 11-NRS for pain documented daily during the last 7 days of the radiation treatment course. Non-inferiority will be tested using a two-sample T-test.

##### Secondary endpoints

Average weekly opioid use will be defined as the average weekly total opioid dose in OMED from randomization to 6 weeks after completion of radiation treatment. Similarly, non-inferiority will be testing using a two-sample T-test.

OMED will be calculated using tables from the Canadian Medical Association Journal [34] and the Canadian Guideline for Safe and Effective Use of Opioids [35].

Duration of opioid requirement will be defined as the time from the start of opioid treatment after date of randomization to the time of stopping opioid analgesia, in days. The average daily 11-NRS for pain will be documented daily from date of randomization to 6 weeks after completion of radiation treatment.

Quality of life will be assessed with EORTC QLQ-C30, and EORTC QLQ-HN 43, before radiation treatment, during the last week of radiation treatment, and 3 months after completion of radiation treatment.

The average weekly opioids dispensed will be defined as the average weekly total opioid dose dispensed by the pharmacy in OMED from randomization to 6 weeks after completion of radiation treatment.

Admissions for any of the following, during radiation or within 3 months of radiation completion will be monitored: febrile neutropenia; other serious infection requiring treatment with intravenous antibiotics; gastrointestinal bleeding attributed to NSAID use; myocardial infarction; stroke; acute kidney injury (defined by the KDIGO Guidelines as: an increase in serum creatinine by ≥26.5 μmol/L within 48 h, OR an increase in serum creatinine to ≥1.5 times the baseline value which is known or presumed to have occurred within the prior 7 days).

Time to feeding tube (e.g. gastrostomy-tube or nasogastric-tube) insertion will be defined as time to feeding tube insertion after randomization, in days. Weight loss from randomization to end of radiation treatment, radiation or chemotherapy treatment interruptions, and death during or within 3 months after completion of radiation treatment will be collected.

Differences between treatment arms for continuous endpoints (e.g. duration of opioid requirement) will be compared using the two-sample T-test for non-inferiority. Differences between treatment arms for categorical or binary end points including CTC-AE rates of grade 2 or higher and admissions will be compared using the Chi-square test or Fisher’s Exact test as appropriate. Differences between treatment arms for time-to-event end points (e.g. time to gastrostomy-tube insertion) will be compared using Kaplan-Meier estimates and the stratified log-rank test (adjusting for stratification by concurrent chemotherapy). Linear mixed effects models will be used to test for non-inferiority between treatment arms for the average daily 11-NRS for pain (from the start of randomization to 6 weeks after completion of radiation treatment). Any tests which meet non-inferiority criteria will be tested for superiority [36].

Subgroup analyses will be performed to assess the primary and secondary endpoints based on the stratification factor (chemotherapy), disease site, and smoking status.

### Ethical considerations

#### Ethics board approval and trial

The PIs have obtained ethical approval and clinical trial authorization from the Western University Health Science Research Ethics Board (Project ID: 115201).

Any modifications to the trial protocol must be approved and enacted by the PI (Current version: 1.0 on July 5, 2020). Protocol amendments will be communicated to all participating centres, investigators, IRBs, and trial registries by the PIs.

#### Informed consent

The consent video as well as the written informed consent form that will be provided to potential study subjects was approved by the IRB/REB and adhere to ICH GCP and the ethical principles that have their origin in the Declaration of Helsinki. Informed consent from each subject prior to beginning any study procedures and treatment(s) will be obtained by the treating physician and confirmed by the hired radiation therapist. Patients enrolled will be informed of all aspects of the study, including the potential risks and benefits involved. They will be given ample time and opportunity to ask questions prior to deciding about participating in the study and be informed that participation in the study is voluntary and that they are completely free to refuse to enter the study or to withdraw from it at any time, for any reason.

The informed consent must be signed and dated by the patient and by the person who conducted the informed consent discussion. A copy of the signed and dated written informed consent form will be given to the patient. The process of obtaining informed consent will be documented in the patient source documents.

#### Confidentiality of subject records and data storage

All data will be stored on REDCap, which is a secure web application for building and managing online databases commonly used in the clinical trials research community [37]. Ongoing auditing will be performed by the LRCP CRU, independent from the trial investigators and sponsor. A confidential patient identification list (Master List) will be maintained throughout the course of the study. All names and identifying information will be kept confidential with access granted to only those involved in direct patient management and in monitoring the conduct of the study.

### Authorship and clinical trial publication

Upon completion of this project, the results will be presented at national and international conferences and subsequently published in a peer-reviewed journal. *Trial* results will remain embargoed until conference presentation of an abstract or until information release is authorized.

Authorship of the trial abstract and ultimately the full manuscript will be decided by the PIs at the time of submission. Professional writers will not be used for either abstract or manuscript preparation.

Final decisions on authorship will be made by the PIs and will be commensurate with the amount of individual contribution, including study design, patient accrual, data analysis and interpretation and manuscript drafting.

Any communication or publication of trial results will be led by the PIs and is expected to occur within 1 year of the primary analysis.

### Financial support for patients

The clinical trial will pay for the cost of medications for a total of $250 per patient. It is possible that the medications may cost more or less than this, depending on the amount of medication the participant may need, or if they have third-party coverage. Participants will only receive $250 regardless of the cost of their medications. This will be paid by cheque, which will be mailed to their home address once the patient is enrolled, randomized, and has been provided with a prescription for analgesic medications. The participants’ address will be obtained from their medical record. It will not be collected.

### Data sharing statement

Deidentified patient data from this trial will not be shared publicly, however, the full manuscript detailing the clinical trial results will be published along with the primary analysis of the outcomes.

## Discussion

Patients with HNC who are undergoing definitive RT with or without chemotherapy suffer significantly from RIM pain [2, 3]. Two prospective studies, although with a limited sample size and mixed results, have assessed the safety and efficacy of different analgesic regimens for RIM pain [38, 39]. In a pilot study, Kataoka et al. randomized 22 stage III or IV patients receiving CRT to Acetaminophen and opioids alone or to Acetaminophen, opioids, and Gabapentin (900 mg daily). They concluded that the addition of Gabapentin conferred no additional analgesic benefit, did not reduce opioid requirements and was associated with worse QoL attributed to weight gain [39]. Conversely, the pilot study conducted by Hermann et al. randomized 60 HNC patients undergoing CRT for stage II-IV disease to either high-dose Gabapentin (2700 mg daily), Hydrocodone and/or Acetaminophen, progressing to Fentanyl as needed, or to low-dose Gabapentin (900 mg daily) with methadone. High-dose prophylactic Gabapentin appeared to reduce opioid requirements, but pain scores worsened throughout treatment irrespective of the analgesic regimen used [38]. No analgesic regimen has yet proven to effectively alleviate RIM pain during RT treatment. It is therefore incumbent upon oncologists to identify effective analgesic methods for RIM pain which improve patient well-being throughout RT, facilitate curative treatment completion by minimizing interruptions, and reduce the risk of chronic opioid use with its associated morbidity and potential mortality.

OPTIMAL-HN is the first randomized clinical trial to assess the efficacy of multimodal analgesia for the management of RIM pain in HNC patients. The multimodal analgesia arm was inspired by the post-operative multimodal analgesia paradigm applied across most surgical specialties in modern practice to reduce post-operative opioid requirements whilst optimizing pain relief [40]. The multimodal analgesia arm in this trial includes Pregabalin, Acetaminophen, and Naproxen, in addition to opioids, if needed. Pregabalin was selected as the Gabapentinoid in this regimen to facilitate compliance given that it is taken twice daily whereas Gabapentin is taken thrice daily. Sixty-two patients will be randomized to either multimodal analgesia or opioid analgesia alone. The non-inferiority of multimodal analgesia to opioid therapy will be determined by pain scores during the last week of RT. Opioid use, duration of opioid requirement, average pain scores during RT, QoL, toxicity, hospitalizations, feeding tube insertions, weight loss, treatment breaks, and death within 3 months of completing RT will also be compared between arms.

The results of OPTIMAL-HN may help establish multimodal analgesia as a new treatment paradigm in the management of RIM pain in HNC patients receiving RT. This paradigm is based on utilizing analgesic medications from different classes and various mechanisms of action with the objective of targeting both the nociceptive and neuropathic components of RIM pain.

### Supplementary Information

**Additional file 1.** Pain Diary.

## Figures and Tables

**Table 3:**
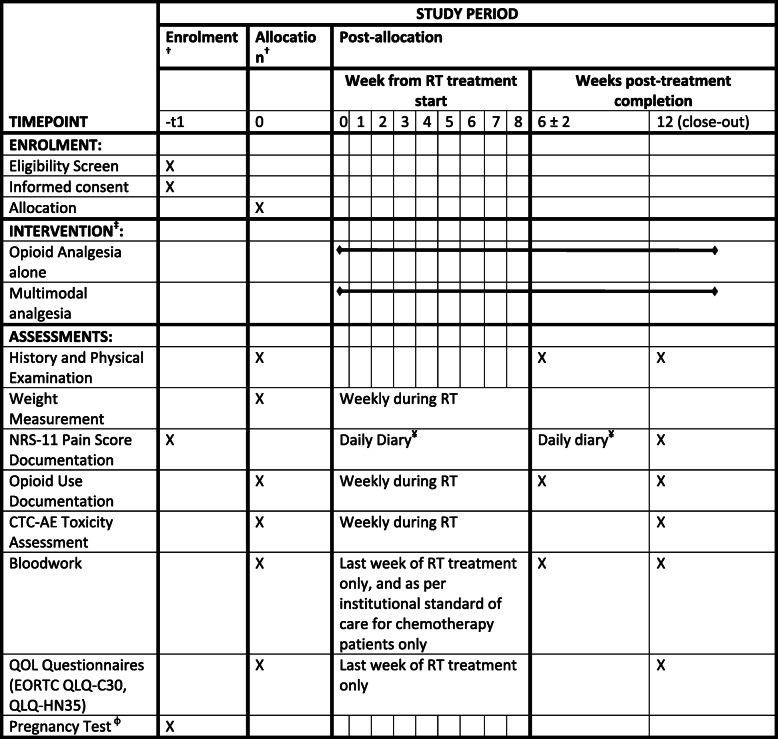
Schedule of enrolment, interventions and assessments

*RT: radiation; 11-NRS: 11-point Numeric Rating Scale for pain*

^**†**^ Enrolment and allocation or randomization occur on the same day during the same patient visit

^**‡**^ The intervention with the analgesic regimen in question, titrated to the patient’s analgesic requirements, will start at the time of randomization, whether that is immediately prior to or during RT treatment, until the final follow-up at 3 months

^ф^ For women of childbearing age only

^**¥**^ From the time of randomization to the 6-week follow-up, patients will be asked to document daily average 11-NRS pain scores

## Data Availability

Not applicable.

## Appendix 2

**Table 4:** World Health Organization tial registration dataset

Item	Description
Primary registry and trial identifying number	ClinicalTrials.gov: NCT04221165
Date of registration in primary registry	January 9, 2020
Secondary identifying numbers	N/A
Source(s) of monetary or material support	PSI Foundation Resident Research Grant
Primary sponsor	Lawson Health Research Institute
Secondary sponsor(s)	N/A
Contact for public queries	Dr. David A. Palma
Contact for scientific queries	Dr. David A. Palma
Public title	Opioid Therapy vs. Multimodal Analgesia in Head and Neck Cancer (OPTIMAL-HN)
Scientific title	Opioid Therapy vs. Multimodal Analgesia in Head and Neck Cancer (OPTIMAL-HN): A Randomized Clinical Trial
Countries of recruitment	Canada
Health condition(s) or problem(s) studied	Head and Neck Cancer Radiation-Induced Mucositis Pain
Intervention(s)	Multimodal Analgesia (Pregabalin, Acetaminophen, Naproxen ± Opioid), or Opioid Analgesia alone
Key inclusion and exclusion criteria	Inclusion Criteria:
	1. Age 18 years or older
	2. Willing to provide consent
	3. Histologically confirmed mucosal head and neck malignancy
	4. Undergoing chemoradiotherapy or radiotherapy alone with a planned total radiation dose of 50 Gy or greater
	5. Eastern Cooperative Oncology Group (ECOG) performance status 0–2
	6. Life expectancy > 6 months
	7. Onset of 4/10 pain on the 11-Numeric Rating Scale that is localized to the mucosa of the mouth or throat, before or during radiation treatment, that is not caused by a current oral candidiasis infection.
	8 Ability to take pills, either by mouth or crushed via nasogastric (NG) tube or gastrostomy (G) tube
	9. Ability to complete the study questionnaires and pain diary
	10. Ability to sign consent without requirement for a substitute decision maker
	Exclusion Criteria:
	1. Skin and salivary gland malignancies
	2. High daily opioid use (defined as 30 mg oral morphine equivalent dose or higher) for more than 7 days at time of enrollment
	3. Concurrent second active malignancy
	4. Pregnant or lactating women
	5. Psychological disorder requiring pharmacologic treatment
	6. Regular systemic steroid use
	7. Regular anticonvulsant or antidepressant use
	8. Renal Impairment
	◦ Defined as creatinine clearance < 60 mL/min
	9. Liver Dysfunction
	◦ Defined as total bilirubin > 34.2 μmol/L
	10. Documented true allergy to Acetaminophen, NSAIDs, Pregabalin or opioids
	11. History of upper gastrointestinal bleed
	12. Known bleeding disorder
	13. History of or current substance use disorder
Study type	Randomized by permuted blocks sequence
	No masking/blinding (open label)
	Parallel 1:1 assignment
Date of first enrolment	August 25, 2020
Target sample size	62
Recruitment status	Recruiting
Primary outcome(s)	Average 11-NRS for pain during last week of radiation treatment
Key secondary outcomes	Average weekly opioid use, duration of opioid requirement, average daily 11-NRS for pain, average weekly opioids dispensed, hospitalizations, time to feeding tube insertion, weight loss, toxicity, treatment interruptions, and death within 3 months of treatment completion.

## Appendix 3

**Table 5:** Multimodal analgesia patient medication schedule

Time	Medication
Upon waking	**Acetaminophen** 1000 mg
	**Pantoprazole** 40 mg
After Breakfast	**Naproxen** 250 mg or 500 mg
	**Pregabalin** at prescribed dose
Lunch	**Acetaminophen** 1000 mg
Supper	**Naproxen** 250 mg or 500 mg
	**Pregabalin** at prescribed dose
At bedtime	**Acetaminophen** 1000 mg

## Abbreviations

11-NRS: 11-Numeric Rating Scale for pain (ranging from 0 to 10)
ADLs: Activities of daily living
AE(s): Adverse event(s)
BID: twice daily
CRO: Contact Research Organization
CRT: Chemoradiotherapy
CRU: Clinical Research Unit
CTC-AE: Common Toxicity Criteria for Adverse Events
DSMC: Data Safety Monitoring Committee
ECOG: Eastern Cooperative Oncology Group
EORTC QLQ-C30: European Organization for Research and Treatment of Cancer Quality of Life Questionnaire Core module
EORTC QLQ-HN 43: European Organisation for Research and Treatment Quality of Life Questionnaire Head and Neck module
HNC: Head and Neck Cancer
ICH: International Council for Harmonisation of Technical Requirements for Pharmaceuticals for Human Use
IRB: Institutional Review Board
KDIGO: The global non-profit organization developing and implementing evidence-based clinical practice guidelines in kidney disease
LRCP: London Regional Cancer Program
NSAID(s): Nonsteroidal anti-inflammatory drug(s)
OMED: Oral morphine equivalent dosing
OPTIMAL-HN: Opioid Therapy vs. Multimodal Analgesia in Head and Neck Cancer
PAiN Relief Regimen: Pregabalin, Acetaminophen, and Naproxen
PI: Principal Investigator
PO: Per os or by mouth
PPI: Proton Pump Inhibitor
PR: Patient Review
QoL: Quality of Life
REB: Research Ethics Board
REDCap: Research Electronic Data Capture, a secure web application for building and managing online databases
RIM: Radiation-induced mucositis
RT: Radiotherapy
SAE(s): Serious adverse event(s)
TID: three times per day
WHO: World Health Organization
