# Supplementary figures and images for: Opioid therapy vs. multimodal analgesia in head and neck Cancer (OPTIMAL-HN): study protocol for a randomized clinical trial
Source: BMC Palliat Care. 2021 Mar 19;20:45. doi: 10.1186/s12904-021-00735-0 (PMC7980584; doi:10.1186/s12904-021-00735-0)

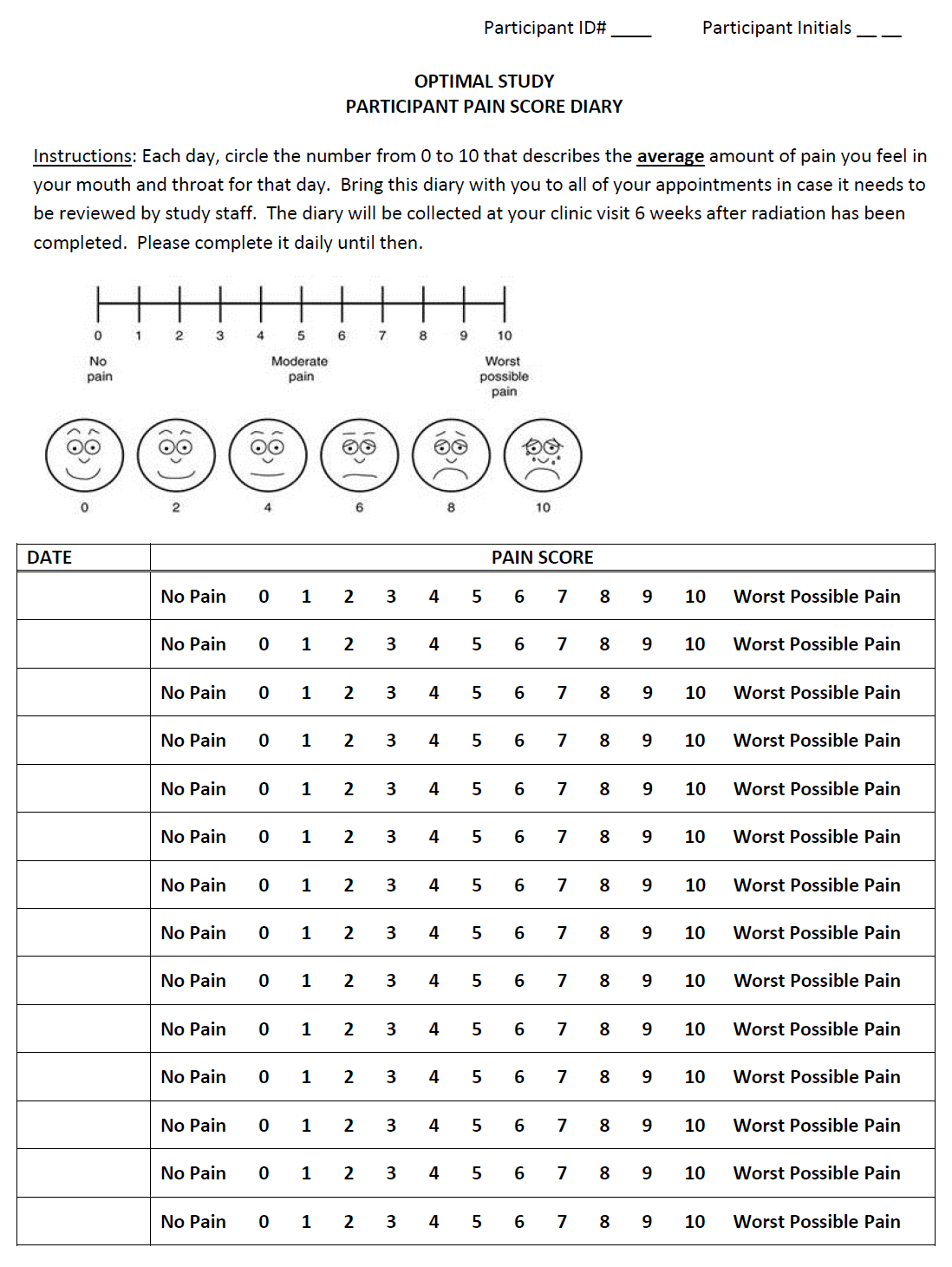

Supplement: Supplementary file 1 — Additional file 1. Pain Diary. [file 12904_2021_735_MOESM1_ESM.docx]
